# Human-Animal Interactions in Dairy Buffalo Farms

**DOI:** 10.3390/ani9050246

**Published:** 2019-05-16

**Authors:** Fabio Napolitano, Francesco Serrapica, Ada Braghieri, Felicia Masucci, Emilio Sabia, Giuseppe De Rosa

**Affiliations:** 1 Scuola di Scienze Agrarie, Forestali, Alimentari ed Ambientali, Università degli Studi della Basilicata, Via dell’Ateneo Lucano 10, 85100 Potenza, Italy; fabio.napolitano@unibas.it (F.N.); ada.braghieri@unibas.it (A.B.); 2 Dipartimento di Agraria, Università degli Studi di Napoli Federico II, Via Università 133, 80055 Portici, NA, Italy; francesco.serrapica83@gmail.com (F.S.); felicia.masucci@unina.it (F.M.); 3 Free University of Bozen-Bolzano, Faculty of Science and Technology, Piazza Università 5, 39100 Bolzano, Italy; emilio.sabia@unibz.it

**Keywords:** dairy buffalo, human-animal relationship, animal behavior, test-retest reliability, avoidance distance, milk production, animal welfare

## Abstract

**Simple Summary:**

The quality of the human-animal relationship plays a central role in determining animal welfare. In this study, we assessed the relationship between stockperson behavior and buffalo behavior. In particular, during milking, we recorded the behavior of stockpeople in terms of quality and quantity of interactions, and we recorded the behavior of animals in terms of restlessness, whereas at the feeding place, we measured the avoidance distance. Avoidance distance of an animal can be defined as the distance to which the animal will allow an unknown person to approach before moving to the side or away. We found that a high percentage of negative stockperson interactions (shouting, talking impatiently, slapping, and handling forcefully) were associated with a high avoidance distance at the feeding place and restlessness during milking. Therefore, appropriate stockpeople training should be conducted to improve the human-animal relationship with positive effects on animal welfare, productivity, and stockpeople safety.

**Abstract:**

This study aimed to assess the relationship between stockperson behavior and buffalo behavior. The research was carried out in 27 buffalo farms. The behavior of stockpeople and animals during milking and the avoidance distance at the feeding place were recorded. Recordings were repeated within one month to assess test-retest reliability. A high degree of test-retest reliability was observed for all the variables with Spearman rank correlation coefficients (*r_s_*) ranging from 0.578 (*p* = 0.002, *df* = 25) for the number of kicks performed during milking to 0.937 (*p* < 0.001, *df* = 25) for the percentage of animals moving when approached by ≤ 0.5 m. The number of negative stockperson interactions correlated positively with the number of kicks during milking (*r_s_* = 0.421, *p* < 0.028, *df =* 25) and the percentage of animals injected with oxytocin (*r_s_* = 0.424, *p* < 0.027), whereas the percentage of negative stockperson interactions correlated positively with the percentage of buffaloes moving when approached at a distance >1 m (*r_s_* = 0.415, *p* < 0.031, *df =* 25). In a subsample of 14 farms, milk yield was correlated positively with the number of positive interactions (*r_s_* = 0.588, *p* < 0.027, *df =* 12) and correlated negatively with the number of steps performed by the animals during milking (*r_s_* = −0.820, *p* < 0.001, *df =* 12). This study showed that the quality of stockpeople interactions may affect buffalo behavior and production.

## 1. Introduction

Over the last few decades, farm animal welfare has become of great interest for the consumers of many different regions, including Europe and North America but also Oceania, Latin America, and Asia [1,2]. As a matter of fact, the consumers’ perceptions of food quality are not only determined by its overall nature and safety but also by the welfare status of the animal from which it was produced [3]. In other words, animal welfare is an important component of an overall “food quality concept”. The human-animal relationship is an important factor when considering farm animal welfare [4,5]. In intensive systems, farm animals are under human control and interact with stockpeople in several situations, including handling and milking. In dairy buffaloes, the farming system has recently become more intensive than it is in dairy cattle, causing potentially higher impacts of human interactions on the animals [6].

The quantity and the quality of these interactions can have distinct outcomes on the emotional state and the cognitive bias of farm animals [4,7]. In addition, there is a large body of evidence suggesting that negative interactions may have a detrimental effect on fertility, growth rates, milk yield, and behavior of farm animals [8,9,10,11]. In buffalo farms, when lactating animals face unfavorable conditions (e.g., stress and lack of habituation to the milking procedures), milk let-down is facilitated by using injections of exogenous oxytocin. Therefore, negative human interactions may increase the use of this practice [12]. Conversely, measures intended to improve this relationship can affect animal reaction to humans [13,14], thus reducing the risk of stockpeople injuries during farm procedures (handling, therapeutic treatments, growth control, loading and unloading from the lorry, etc.). Moreover, beneficial effects of gentling on growth of calves and veal quality have been documented [11,15]. 

The avoidance distance of animals to humans has been widely used as a measure of the quality of the human-animal relationship [5,10,16,17,18]. Avoidance distance of an animal is the distance to which the animal will allow an observer to approach before it moves to the side or away [5,19]. The rationale behind this measure is that the lower the distance between animal and observer is, the lower the level of fear towards humans is [5,16]. In cattle and buffaloes, the avoidance distance has been measured both in the barn and at the feeding place [17,18,20,21], and, at least in cattle, these two measures have proven to be highly correlated [17,21]. Therefore, due to feasibility reasons, the avoidance distance at the feeding place was used in the Welfare Quality^®^ assessment protocol for cattle [22] and buffaloes [23]. Previously, De Rosa et al. [23] reported a high inter-observer reliability of the avoidance distance at the feeding place, with a Spearman coefficient of 0.92, whereas no information was available on test-retest reliability (i.e., consistency of the measurement when repeated within a certain period of time) of the avoidance distance at the feeding place and animal and stockpeople behaviors in the milking parlor.

In addition, while previous investigations conducted on dairy cattle [5], pigs [24], and poultry [25] found significant correlations between stockpeople behavior, animal behavior, and productive parameters such as milk yield, milk quality, and growth rate, little is known about these relationships in dairy buffaloes [12]. In particular, no information is available on the relationship between the behavior of buffaloes in the milking parlor and their reaction to an approaching person at the feeding place (i.e., avoidance distance) or on the relationship between the quality of human-animal relationships and milk production.

Therefore, the present study aimed to assess the test-retest reliability of the stockperson and buffalo behavior in the milking parlor and the test-retest reliability of the response of buffaloes when approached at the feeding place. Then, within the frame of the human-animal relationship model as set by Hemsworth [26], the relationships between human behavior in the milking parlor, animal behavior in the milking parlor, buffalo reactivity to an unknown person at the feeding place, and milk production was studied. In addition, the effect of the milking parlor design on human and buffalo behaviors was studied.

## 2. Methods

### 2.1. Farms, Animals, and Procedure

The research was carried out in 27 Italian buffalo farms located in Campania (n = 18) and Apulia (n = 9) regions. The herd size ranged from 90 to 1,400 head (number of buffalo cows ranging from 46 to 930), with lactating buffalo ranging from 20 to 300. Fourteen farms were equipped with herring-bone parlors, whereas the remaining used tandem parlors. Observations were conducted from September 2014 to February 2015 by two trained assessors. They were trained in buffalo farms not involved in the experiment for identifications of stockperson and animal behaviors through direct observations. The human-animal relationship was assessed by performing two different methods—avoidance distance at the feeding place 5 min after morning feed distribution and behavioral observations of stockperson and animals during afternoon milking. The time interval between the two tests—albeit variable, as it was dependent on farm routine practices (feeding routine and milking routine, respectively)—ranged from 5 to 7 h. These tests were repeated within one month to assess test-retest reliability; before starting the second observation session, the observers verified that no major changes had occurred in farm management. However, the animals observed in the two visits were not exactly the same. In all farms, stockperson and buffalo behaviors in the milking parlor were recorded by the same observer, whereas the stockperson’s behavior when moving the animals from the waiting area to the milking parlor was always observed by the second observer. The latter also measured the avoidance distance at the feeding place. A management questionnaire aimed to gather information about herd size, milking routine, farm management, housing, and milking parlor characteristics was administered by the observer to farm owners during the first visit. In all farms, testing order was always the same: avoidance distance at the feeding place (morning,), filling questionnaire (morning), and behavioral observations during afternoon milking. Fourteen farms were enrolled in the national milk recording scheme. Therefore, for these farms, the data on milk production (expressed as kg/head/year) and milk quality (in terms of percentage of fat and protein) were collected. 

### 2.2. Avoidance Distance at the Feeding Place

Five minutes after feed distribution, the avoidance distance at the feeding place was measured. The number of animals tested in each farm ranged from 20 to 100. The test was conducted according to the procedure reported by Waiblinger et al. [21]. The observer waited for the individual buffalo to look at him before approaching the animal. Animals were approached by the test person in a standardized way, i.e., directly from the front, starting, whenever possible, from a distance of 2 m, walking slowly (around one step per second), looking at the animal muzzle without staring at the buffalo’s eyes, and keeping an arm at an angle of about 45° in front of the body. The test was ended whenever the animal withdrew (i.e., taking steps away from the observer or turning the head more than 45°). If the buffalo cow accepted the touch on the muzzle/nose, the experimenter tried to stroke the cheek of the animal for at least 1 s but not longer than 3 s. Avoidance distance was estimated at the moment of buffalo cow withdrawal as the distance between the observer’s hand and the animal’s head with a resolution of 10 cm. The distance was measured by counting the steps of the observer and converting into meters by measuring the length of the observer’s step. In a case of withdrawing at the moment of touching the nose or muzzle, an avoidance distance of 10 cm was recorded, whereas a distance of 0 cm was assigned when the animal allowed itself to be touched and stroked. Animals were consecutively tested, but, in order to reduce the risk of influencing the neighbor’s behavior, every second animal was tested. For each farm and each visit, the mean avoidance distance at the feeding place was calculated. In addition, the following variables were calculated, as reported in the Welfare Quality protocol for buffaloes [23]:

animals moving at a distance >1 m, %;
animals moving at a distance ≤1 m and ≥0.5 m, %;
animals moving at a distance ≤0.5 m, %;
animals that can be touched, %.

### 2.3. Stockperson and Buffalo Behavior during Milking

Stockperson’s behavior was observed from moving the animals to the waiting area to the exit from the milking parlor. A total of 55 stockpeople were observed. Before starting the second observation session, the observers verified that no change in the personnel had occurred. We defined the interactions promoting social partnership as positive, the interactions showing a dominant role of the stockperson towards the animals as neutral, and those involving harsh physical or verbal interplay as negative. Therefore, the variables concerning the stockpeople behavior were classified and recorded as follows: number of positive (talk quiet, pet, touch gentle), neutral (talk dominant, hand gentle, stick gentle) and negative (shout, talk impatient, stick, slap, and handle forceful) interactions, as indicated by Waiblinger et al. [5]. The percentages of these three variables in relation to the total of interactions were also calculated. For each farm and each visit, the average number of interactions per milked buffalo was calculated. If two persons milked together, the sum of both was used to calculate this average. The occurrence of oxytocin injection at milking (number of injected animals/number of observed animals) was also recorded.

The buffalo behaviors, recorded from the entrance in the milking parlor to the removal of the milking cluster, were step (foot lifted less than 15 cm off of the ground) and kick (raised above 15 cm off of the ground, even if a clear kick was not visible). They were registered whenever the stockperson was within 0.5 m of the animals. 

### 2.4. Statistical Analysis

Data were analyzed with the Statistical Analysis Systems Institute package [27]. The farm was used as the experimental unit. Therefore, for each visit, the values of the behavioral variables concerning both the animals and the stockpeople were averaged within farms. In order to avoid non-independent results, the number and the percentage of neutral interactions and the percentage of animals moving at a distance ≤1 m and ≥0.5 m were excluded from the analyses, as they corresponding to intermediate, thus less informative, categories. Then, the means were used to calculate the test-retest reliability of the variables concerning the behaviors of the stockperson (number of positive and negative interactions/milked buffalo, percentage of positive and negative interactions, percentage of animals injected with oxytocin) and the animals (number of steps and kicks/milked buffalo, the avoidance distance at the feeding place, percentage of animals that can be touched, percentage of animals moving at a distance ≤0.5 m, percentage of animals moving at a distance >1 m). Test–retest reliability was calculated using the Spearman rank correlation test (*r_s_*). Limits of agreement were also calculated to test whether bias existed between visits [28]. Subsequently, these variables were averaged within the farm across the two visits. These farm averages were used to compute the correlations between stockpeople behavior variables, including the number of milked buffaloes/stockperson, and animal behavior variables using the Spearman rank correlation test. In addition, only for the 14 farms enrolled in the national milk recording scheme, we calculated the correlation between milk production and the stockpeople behavior variables as well as the correlation between milk production and the animal behavior variables using the Spearman rank correlation test.

The Kruskal-Wallis one-way ANOVA test was used to assess the effect of milking parlor design (herring-bone: n = 14 and tandem: n = 13) on the variables collected on stockpeople and animals.

## 3. Results and Discussion

### 3.1. Test-Retest Reliability

Table 1 and Table 2 indicate the test-retest reliability of the variables concerning the behavior of stockpeople and animals, respectively. According to Martin and Bateson [29], a satisfactory threshold for correlation coefficients may be considered 0.7, as roughly 50% of variance in one set of observations is explained by the other set of observations. In our study, the reliability of the variables measured on the stockpeople may be considered satisfactory, with *r_s_* value above 0.7 for most of them, whereas the number of positive interactions and the percentage of negative interactions were 0.650 and 0.677, respectively; *p* < 0.001, *df =* 25. However, the values of these coefficients were lower than those obtained for the animal-based variables, which ranged from 0.578 (*p* = 0.002, *df* = 25) for the number of kicks performed by the animals during milking to 0.937 (*p* < 0.001, *df* = 25) for the percentage of animals that moved when approached by ≤0.5 m. Limits of agreement mostly confirm the results expressed in terms of *r_s_*. In cattle, high long-term consistency (farm visits were conducted at bimonthly intervals) was observed by Winckler et al. [30] for both avoidance distance measured in the barn and at the feeding place, whereas moderate to high test-retest reliability (recorded at 2–3 week intervals) was observed in buffaloes for avoidance distance measured in the barn [20]. Our results indicate that the avoidance distance at the feeding place and stockpeoples’ and animals’ behaviors during milking can be reliably used as indicators of the quality of the human-animal relationship, as also suggested by Hemsworth et al. [16] and Waiblinger et al. [21] for dairy cows.

### 3.2. Stockperson Behavior

The median and the range of stockperson behavioral variables are shown in Table 3. The behavior of the stockpeople was characterized by a low number of interactions with the animals. These interactions were mainly neutral, whereas negative interactions were the lowest, with a high degree of variability among farms. Although Breuer et al. [10] and Hemsworth et al. [16] used different categories to classify human-animal interactions in cattle, if the definitions are considered, these categories roughly correspond to those used in the present study (i.e., positive, negative, and very negative interactions from Breuer et al.’s [10] and Hemsworth et al.’s [16] studies corresponding to positive, neutral, and negative interactions, respectively, in our study). These authors recorded a similar total number of interactions. Conversely, Waiblinger et al. [5] and Ivemeyer et al. [31] reported a higher number of interactions. However, this discrepancy is likely due to the fact that the latter authors recorded the stockpeople behavior since the animals were moved from the barn, whereas in this and the other previously mentioned studies, the behavior of stockpeople was observed when the animals were moved from the waiting area to the milking parlor until their exit from it. Percent negative interactions showed the lowest value in this study, as in all the previous studies conducted on dairy cattle, whereas neutral and positive interactions showed the highest and the intermediate percentages, respectively. Although neutral interactions were the most represented in a study conducted by Saltalamacchia et al. [12] in a previous work on dairy buffaloes, these authors recorded a higher number of negative as compared to positive interactions. We can hypothesize that the attitude and consequently the behavior of the stockpeople working in buffalo milking parlors has improved over the last decade. Negative interactions are able to increase the level of fear of humans, whereas positive interactions can decrease it with more beneficial effects on animal welfare as compared with neutral interactions [4].

The design of the milking parlor affected only the number (median, range: 0.09, 0.03–0.64 versus 0.01, 0.00–0.37, respectively) and percentage (15.00, 4.71–87.88 versus 2.56, 0.00–32.35, respectively) of negative interactions (*χ^2^* = 7.26, *p* = 0.007 and *χ^2^* = 5.75, *p* = 0.016, respectively, *df =* 1) with higher levels of negative interactions in tandem parlors as compared with herring-bone parlors. Although in buffaloes, no studies about the effect of milking parlor design on human-animal interaction are available to support our hypothesis, we postulate that tandem parlors require animals to be individually handled in order to let them in and out of each stall, whereas in herring-bone parlors, animals are handled in groups, which may facilitate their entrance and exit with a reduced likelihood of negative interactions because of reduced handling and individual interactions. In addition, the type of parlor tended to influence the percentage of animals injected with oxytocin (*χ^2^* = 3.32, *p* = 0.067) with lower percentages in herring-bone parlors as compared with tandem parlors (6.55, 0.00–27.81 versus 14.21, 0.00–100, respectively).

### 3.3. Buffalo Behavior and Production

The median and the range of buffalo behavioral variables are shown in Table 4. Animal restlessness at milking may be caused by many different factors, such as pushing of adjacent cows (only in herringbone parlors), lameness, presence of hematophage insects, poor maintenance of milking machine, etc. However, it is widely accepted that at least a component of these behavioral expressions is related to the quality of the human-animal relationship. In this study, the number for stepping was in line with that reported in some studies conducted on dairy cows [16,31] but lower than that reported in other studies conducted in dairy buffaloes [12] and cattle [5,10]. Conversely, the number for kicking was higher than that reported in all the previously cited articles on cattle, albeit it was lower than that observed in buffaloes [12]. This may be due to a higher sensitivity and reactivity of buffaloes to the milking routine, as also suggested by the high number of animals injected with oxytocin observed in this study and in previous studies conducted at farms [12] and individual levels [32] as compared with cattle [33].

In this study, the avoidance distance at the feeding place was lower than that reported by Shahin et al. in dairy cattle (mean = 52 cm) [34], higher than those measured in fattening bulls (mean = 12–15 cm) [18] and in dairy cattle (median = 8–10 cm) [35], and comparable to those reported by De Rosa et al. [23] for buffaloes (median = 20 cm) and by Windschnurer et al. [17] (median = 18.0 cm) and Battini et al. in dairy cattle (mean = 25) [36]. A previous study also reported a lower avoidance distance in buffaloes as compared with cattle kept in the same management and housing conditions [20].

Only the percentage of buffaloes moving when approached at a distance >1 m was affected by the design of the milking parlor, with tandem milking parlors showing a percentage of buffaloes moving when approached at a distance >1 m higher than herring-bone milking parlors (*χ^2^* = 5.06, *p* = 0.024, *df* = 1, median = 2.67, range = 0.00–52.78 versus 0.00, 0.00–11.90, respectively). These results soundly match those on stockperson behavior, where the tandem milking parlor induced a higher level of negative interactions and tended to increase the number of animals injected with oxytocin.

### 3.4. Correlating Stockperson and Buffalo Behaviors

Negative stockperson interactions—both in terms of absolute number and percentage—correlated positively with the number of kicks during milking (*r_s_* = 0.421, *p* = 0.028 and *r_s_* = 0.430, *p* = 0.025, respectively; *df =* 25), whereas only the number of negative interactions correlated positively with the percentage of animals injected with oxytocin (*r_s_* = 0.424, *p* = 0.027, *df =* 25). These results are consistent with the hypothesis that the stockperson behavior can influence animal behavior during milking [5,16] and indicate that a negative behavior expressed by stockpeople at the time of milking can have a detrimental effect on buffalo cows. In our study, buffaloes, concomitant to a negative human approach, displayed higher levels of restlessness in terms of number of kicks, with an increased number of animals injected with oxytocin due to either impaired milk let down or stockpeople willing to speed up the milking routine. The number of negative stockperson interactions tended to be correlated positively with the percentage of buffaloes moving when approached at a distance >1 m (*r_s_* = 0.355, *p* = 0.069, *df =* 25), whereas the percentage of negative stockperson interactions was correlated positively with the percentage of buffaloes moving when approached at a distance >1 m (*r_s_* = 0.415, *p* = 0.031). This result suggests that buffaloes, as with other farm animals, are able to generalize their response to humans, and if they perceive negative stimuli from the stockpeople in the parlor, they also tend to increase their avoidance response to an unknown person approaching them at the feeding place. The percentage of buffaloes moving when approached at a distance >1 m also tended to be correlated positively with the number of lactating animals per stockperson (*r_s_* = 0.345, *p* = 0.078, *df* =25), which may indicate that a high animals to milkers ratio may impair the establishment of a positive human-animal relationship and reduce the confidence of the animals towards humans. A previous study primarily conducted on dairy cattle found no association between the number of animals per farm and the level of animal welfare [37] and suggested that efforts should concentrate on the improvement of animal welfare independently from the size of the farms. However, in the present study, the effect of the ratio of animal to milker was investigated (rather than the effect of the number of animals per farm), and a high ratio, while increasing the work load for stockpeople, may have potentially negative consequences on the quality of the human-animal relationship. The percentage of positive stockperson interactions tended to be correlated negatively with the percentage of animals injected with oxytocin (*r_s_* = −0.343, *p* = 0.080, *df =* 25). 

The milk production per animal per year was correlated negatively with the number of steps (*r_s_* = 0.820, *p* < 0.001, *df =* 12) and correlated positively with the number of positive stockperson interactions (*r_s_* = 0.588, *p* = 0.027, *df =* 12). This latter finding suggests that positive stockperson interactions may improve the quality of human-animal relationships and increase the welfare of the animals with beneficial effects on milk production, as also reported by other authors in previous studies on dairy cattle (e.g., [16]). However, these results should be taken with caution, as they are based on a limited number of farms (n = 14, i.e., only those adhering to the official Italian recording system).

## 4. Conclusions

The present study confirmed that the test-retest reliability of the variables used to assess the human-animal relationship in buffaloes was high. Also relevant are the findings showing the relationship between negative stockperson behavior and the reaction of buffalo cows in terms of restlessness and, possibly, consequent impaired milk let down. Therefore, appropriate stockpeople training should be conducted to improve human-animal relationships with positive effects on animal welfare, productivity, and stockpeople safety. In addition, the correlation between the percentage of negative stockperson interactions at milking and the percentage of animals moving when approached at a distance >1 m showed that buffalo cows are able to generalize their responses to humans, and if they perceive negative stimuli from the stockpeople in the parlor, they also tend to increase their avoidance response to an unknown person approaching them at the feeding place. Therefore, the avoidance distance of buffaloes at the feeding place is a promising variable to be used for the assessment of the quality of human-animal relationships, as demonstrated in other animal species. Although based on a limited number of farms, also relevant were the results displaying a correlation between stockperson positive interactions and milk production. 

Stockpeople behavior was also affected by milking parlor design, with higher negative interactions in tandem parlors than in herring-bone parlors, possibly due to the fact that animals had to be individually handled to let them in and out of each stall. In farms equipped with tandem parlors, the percentage of buffaloes reacting at more than 1 m to an approaching human was higher, and the percentage of animals injected with oxytocin tended to increase.

## Figures and Tables

**Table 1:** Test–retest reliability of the variables observed on the stockpeople using Spearman rank correlation coefficient (*r_s_*) and limits of agreement (mean of differences ±2 SD). Calculations were based on two farm visits (n = 27).

	Spearman Statistics		Limits of Agreement		
Variable	*r_s_*	*p*-Value	Mean	Mean +2 SD	Mean −2 SD
Positive interactions/milked buffalo, n	0.650	<0.001	−0.01644	0.30416	−0.33703
Negative interactions/milked buffalo, n	0.825	<0.001	0.002949	0.33771	−0.33181
Positive interactions, %	0.771	<0.001	0.641434	34.20647	−32.9236
Negative interactions, %	0.677	<0.001	0.718693	23.07633	−21.6389
Animals injected with oxytocin, %	0.799	<0.001	2.616128	22.58423	−17.352

**Table 2:** Test–retest reliability of the variables observed on the animals using the Spearman rank correlation coefficient (*r_s_*) and limits of agreement (mean of differences ±2 SD). Calculations were based on two farm visits (n = 27).

	Spearman Statistics		Limits of Agreement		
Variable	*r_s_*	*p*-Value	Mean	Mean +2 SD	Mean −2 SD
Steps/buffalo, n	0.900	<0.001	0.079351	1.385843	−1.22714
Kicks/buffalo, n	0.578	0.002	0.007591	0.682179	−0.667
Animals moving at a distance >1 m, %	0.821	<0.001	−0.52111	6.873861	−7.91608
Animals moving at a distance ≤0.5 m, %	0.937	<0.001	0.051073	16.34284	−16.2407
Animals that can be touched, %	0.903	<0.001	−0.68902	16.08882	−17.4669
Median avoidance distance at the feeding place, m	0.923	<0.001	0.013462	0.135306	−0.10838

**Table 3:** Median and range of the variables observed on the stockpeople (n = 27).

Variable	Median	Range
Positive interactions/milked buffalo, n	0.15	0–0.79
Neutral interactions/milked buffalo, n	0.36	0.03–3.28
Negative interactions/milked buffalo, n	0.04	0–0.64
Positive interactions, %	19.64	0–83.88
Neutral interactions, %	61.29	9.09–93.88
Negative interactions, %	7.61	0–87.88
Animals injected with oxytocin, %	9.75	0–100
Milked buffalo/stockperson, n	55.50	19.12–100

**Table 4:** Median and range of the variables observed in the animals (n = 27).

Variable	Median	Range
Steps/milked buffalo, n	0.88	0.11–6.62
Kicks/milked buffalo, n	0.22	0–0.84
Animals moving at a distance >1 m, %	0	0–52.78
Animals moving at a distance ≤1 m and ≥0.5 m, %	7.32	0–46.30
Animals moving at a distance ≤0.5 m, %	49.31	0.93–100
Animals that can be touched, %	34.17	0–77.38
Median avoidance distance at the feeding place, m	0.23	0.04–1.17
Milk production ^1^, kg/head/year	1995	1593–2540

^1^ This variable was measured only in 14 farms enrolled in the national milk recording scheme.
